# Downregulation of BTLA on NKT Cells Promotes Tumor Immune Control in a Mouse Model of Mammary Carcinoma

**DOI:** 10.3390/ijms19030752

**Published:** 2018-03-07

**Authors:** Divya Sekar, Luisa Govene, María-Luisa del Río, Evelyn Sirait-Fischer, Annika F. Fink, Bernhard Brüne, José I. Rodriguez-Barbosa, Andreas Weigert

**Affiliations:** 1 Institute of Biochemistry I, Faculty of Medicine, Goethe-University Frankfurt, 60590 Frankfurt, Germany; sekar@biochem.uni-frankfurt.de (D.S.); luisa.govene@gmx.de (L.G.); sirait@biochem.uni-frankfurt.de (E.S.-F.); fink@biochem.uni-frankfurt.de (A.F.F.); b.bruene@biochem.uni-frankfurt.de (B.B.); 2 Transplantation Immunobiology Section, School of Biological Sciences and Biotechnology, University of Leon and Castilla and Leon Regional Transplantation Coordination, Leon University Hospital, 24071 Leon, Spain; m.delrio@unileon.es (M.-L.d.R.); ignacio.barbosa@unileon.es (J.I.R.-B.); 3 Project Group Translational Medicine and Pharmacology TMP, Fraunhofer Institute for Molecular Biology and Applied Ecology, IME, 60590 Frankfurt, Germany

**Keywords:** natural killer T cells, inflammation, cancer

## Abstract

Natural Killer T cells (NKT cells) are emerging as critical regulators of pro- and anti-tumor immunity, both at baseline and in therapeutic settings. While type I NKT cells can promote anti-tumor immunity, their activity in the tumor microenvironment may be limited by negative regulators such as inhibitory immune checkpoints. We observed dominant expression of B- and T-lymphocyte attenuator (BTLA) on type I NKT cells in polyoma middle T oncogene-driven (PyMT) murine autochthonous mammary tumors. Other immune checkpoint receptors, such as programmed cell death 1 (PD-1) were equally distributed among T cell populations. Interference with BTLA using neutralizing antibodies limited tumor growth and pulmonary metastasis in the PyMT model in a therapeutic setting, correlating with an increase in type I NKT cells and expression of cytotoxic marker genes. While therapeutic application of an anti-PD-1 antibody increased the number of CD8+ cytotoxic T cells and elevated IL-12 expression, tumor control was not established. Expression of ZBTB16, the lineage-determining transcription factor of type I NKT cells, was correlated with a favorable patient prognosis in the METABRIC dataset, and BTLA levels were instrumental to further distinguish prognosis in patents with high ZBTB16 expression. Taken together, these data support a role of BTLA on type I NKT cells in limiting anti-tumor immunity.

## 1. Introduction

The tumor-associated immune system is a critical determinant of patient survival and therapy success. Density and activity of cytotoxic lymphocytes such as γδ T cells or CD8+ T cells are associated with favorable prognosis, whereas the presence of suppressive myeloid cells such as macrophages or myeloid-derived suppressor cells is often a marker of poor prognosis [1]. Thus, certain tumor immune profiles are desirable. This is not only true at baseline, but also following cancer therapy as demonstrated by the recent success of immune checkpoint inhibitors that yield a survival benefit in specific tumor entities [2]. Immune checkpoints are receptor ligand interactions that determine propagation versus termination of immune responses. Antibody-mediated blockade of immune inhibitory checkpoints using neutralizing antibodies that disrupt, e.g., the interaction of programmed cell death 1 (PD-1) on lymphocytes with programmed death-ligand 1 (PD-L1), which is upregulated as a negative feedback following lymphocyte activation to terminate inflammation, is a recently validated approach for immune intervention. Therefore, immune checkpoint inhibition releases the brakes on tumor immunity and reactivates anti-tumor immune responses [2]. Besides PD-1, other immune checkpoint receptors exist, whose therapeutic potential needs to be explored. Among them is B and T lymphocyte attenuator (BTLA, CD272), a co-inhibitory receptor with an intracellular immunoreceptor tyrosine-based inhibitory motif (ITIM), belonging to the immunoglobulin superfamily with a pattern of expression restricted to hematopoietic cells [3]. BTLA binds to its ligand, the herpesvirus entry mediator (HVEM, TNFSF14), a member of the tumor necrosis factor receptor superfamily that is expressed in both hematopoietic cells (T cells, B cells, NK cells, DCs, and myeloid cells) and non-hematopoietic cells [4]. High BTLA expression is observed on B cells during steady-state, whereas conventional T cells, NKT cells, NK cells and dendritic cells express low levels [5]. Upon activation, BTLA expression on B cells decreases, while it increases on T cells, especially on Th1 cells [3,6]. There is an inverse correlation between BTLA and HVEM expression upon T cell activation, which promotes *trans* interactions as opposed to *cis* interactions in resting T cells [7]. While BTLA may promote T cell survival, it decreases proliferation and activity, thereby promoting peripheral tolerance, but limiting anti-tumor immunity [8]. Besides regulating the activity of adaptive immune cells, BTLA also interferes with innate or innate-like lymphocytes. It has been proposed as a potent inhibitory receptor on γδ T cells [9], and the severe immunopathology associated to Con A-induced liver damage in BTLA-deficient mice was largely traced back to its inhibitory role on cytokine production by type I NKT cells [10]. 

NKT cells are thymus-derived innate-like T cells that express NK1.1 and T cell receptors, therefore featuring characteristics and function of both NK cells and conventional T cells [11]. While conventional T cells recognize peptide antigens presented in the context of MHC class I or class II molecules, NKT cells recognize self- and foreign lipid antigens presented via CD1 molecules (a non-polymorphic MHC class I-like molecule). CD1 molecules (CD1d in the mouse, CD1A-E in humans) are typically expressed by antigen-presenting cells (APCs). Interaction between the NKT TCR and the antigen-CD1d complex leads to a rapid activation of the NKT cells, which release a large amount of inflammatory cytokines due to their memory-like phenotype (CD69 and CD44 expression) [12]. Within this population of CD1d-restricted T cells, different subsets can be distinguished. NKT type I, also called invariant NKT cells or iNKT, express an invariant TCR α chain with a Vα14 Jα18 gene segment in mice (Vα24 Jα18 in humans) and a limited number of TCRβ chains. They are further defined by their ability to recognize CD1-bound α-galactosylceramide (α-GalCer), a glycolipid antigen isolated from marine sponges, and its derivatives [13]. In contrast, type II NKT cells show a more diverse pattern of TCR usage and recognition of lipid antigens [14]. In tumors, opposing functions have been attributed to type I versus type II NKT cells. While type I NKT cells promote tumor immunosurveillance by direct cytotoxicity towards tumor and other cells or the release of immunostimulatory cytokines such as interferon-γ (IFN-γ) or granulocyte-macrophage colony-stimulating factor (GM-CSF), type II NKT cells actively hinder anti-tumor immunity by promoting the accumulation of suppressive myeloid cells [15,16]. Activation of type I NKT cells in tumors therefore appears desirable, since they display direct cytotoxicity towards tumor cells and produce large amounts of IFN-γ to further activate other cytotoxic immune cells such as NK cells and CD8+ T cells. Consequently, several clinical trials are under way to harness the anti-tumor potential of type I NKT cells [14,17]. Strategies include direct application of α-GalCer, adoptive transfer of APCs loaded with α-GalCer and adoptive transfer of ex-vivo expanded NKT cells themselves. In light of these trials, the possibility of functional suppression of existing or newly expanded NKT cells in the tumor microenvironment, e.g., via immune checkpoints, needs to be investigated. In this study, we therefore analyzed the expression of immune checkpoint receptors PD-1 and BTLA on NKT cells in a model of mammary carcinoma and explored the potential of downregulating BTLA expression on type I NKT cells and the consequences in tumor progression and the propagation of metastasis.

## 2. Results

### 2.1. Type I NKT Express BTLA in PyMT Mammary Tumors

**Figure 1:**
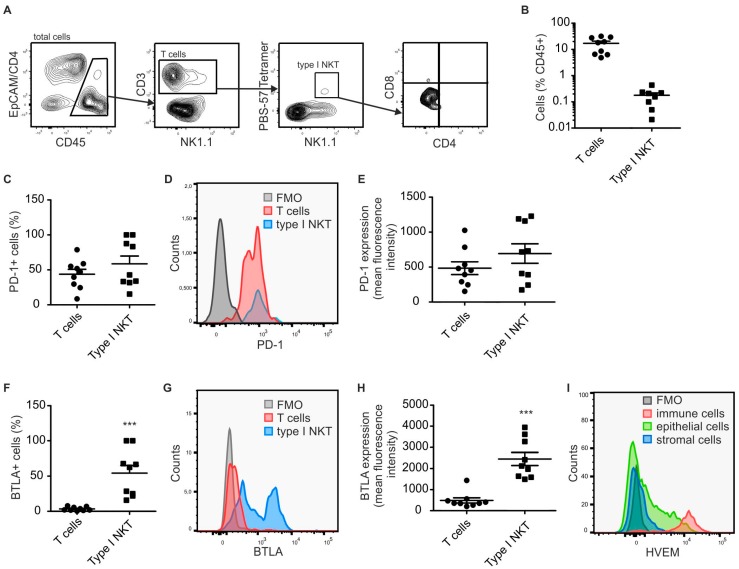
Type I NKT cells express high levels of BTLA in PyMT tumors. PyMT tumors (*n* = 9) were harvested and single cell suspensions were analyzed by flow cytometry. (**A**) Gating strategy used to identify total CD3+ T cells and PBS-57-CD1d tetramer reactive type I NKT cells in PyMT tumors. (**B**) The relative abundance of total CD3+ T cells and PBS-57-CD1d tetramer reactive type I NKT cells in the total CD45+ immune cell pool in PyMT tumors is shown. Data are means + SEM. (**C**–**E**) Relative abundance (**C**); and expression levels (**D**,**E**) of PD-1 on T cell populations are displayed. (**F**–**H**) Relative abundance (**F**); and expression levels (**G**,**H**) of BTLA on T cell populations are displayed. (**I**) Representative FACS histogram showing the expression of HVEM on different cells in PyMT tumors. *p*-values were calculated using two-tailed Student’s *t* test, *** *p* < 0.001.

To analyze expression of immune checkpoint receptors on tumor-infiltrating lymphocytes, with a focus on NKT cells, we performed FACS analysis of single cell suspensions derived from murine PyMT mammary tumors at Week 18. The PyMT model is driven by the expression of the polyoma middle T oncoprotein in the mammary epithelium and recapitulates human HER2+ metastatic breast cancer [18]. At Week 18, PyMT tumors in C57BL/6 mice have usually progressed to a pre-metastatic state. To specifically identify type I NKT cells, PBS-57-loaded CD1d tetramers were used. As expected, type I NKT comprised a minor population of tumor infiltrating immune cells, whereas conventional T cells were highly abundant (Figure 1A,B). When analyzing expression of immune checkpoint receptors, type I NKT showed a very distinct pattern compared to conventional T cells. While expression of immune checkpoint receptors such as PD-1 (Figure 1C–E) was comparable, abundant expression of BTLA was only observed on type I NKT cells (Figure 1F–H). HVEM, the ligand of BTLA, was expressed at high levels on immune cells in PyMT-tumors and at lower variegated levels on tumor epithelial cells (Figure 1I), allowing the engagement of BTLA by several cells in the microenvironment of PyMT tumors.

### 2.2. Downregulation of BTLA But Not PD-1 Blockade Reduces PyMT Mammary Tumor Progression

**Figure 2:**
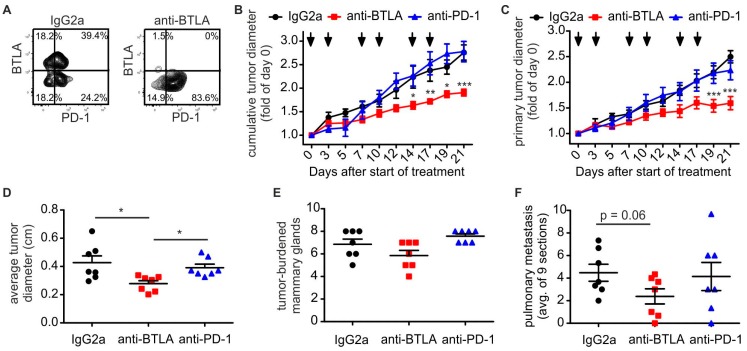
Anti-BTLA antibodies reduce PyMT tumor growth. PyMT tumors were treated with 5 mg/kg of each anti-BTLA, anti-PD-1 or isotype control (IgG2A) antibodies (*n* = 7 each) twice weekly for three weeks once the first tumor reached a size of 0.5 cm in diameter. (**A**) Representative FACS plots show expression of BTLA on T cells after treatment with anti-BTLA or isotype control antibody. (**B**,**C**) Cumulative tumor diameters (**B**); and the diameter of the primary tumor (**C**) over time are shown. (**D**–**F**) The endpoint, the average tumor diameter per mammary gland (**D**); the number of tumor-burdened mammary glands (**E**); and the occurrence of pulmonary metastasis (**F**) are displayed. *p*-values were calculated using two-way ANOVA (**B**,**C**) or one-way ANOVA (**D**–**F**) with Bonferroni’s correction, * *p* < 0.05, ** *p* < 0.01, *** *p* < 0.001.

Given the role of type I NKT cells in anti-tumor immunity, we wondered whether inhibiting BTLA signaling would affect tumor progression in the PyMT model. To this end, we subjected PyMT mice to BTLA or PD-1 immune checkpoint blockade in a therapeutic setting, once the first tumor had reached a diameter of 0.5 cm. Mice received i.p. injections with either a BTLA-blocking antibody [5], a PD-1-blocking antibody [19], or an isotype control (rat IgG2a) twice weekly for three weeks. Efficacy of the BTLA antibody, which has been shown to downregulate BTLA expression [5], was confirmed by flow cytometry (Figure 2A). Interestingly, while the anti-PD1 antibody did not slow tumor progression in the PyMT model, despite the high level of PD-1 expression on tumor-infiltrating T cells, the anti BTLA antibody markedly decreased tumor growth, when looking at the overall tumor burden (Figure 2B), the primary tumor (Figure 2C) or average tumor size (Figure 2D). In contrast, the total number of tumor-carrying mammary glands was not different among the experimental groups (Figure 2E). When investigating pulmonary metastasis, the anti-BTLA antibody showed a strong tendency to decrease metastasis burden, whereas the anti-PD-1 antibody was again inefficient (Figure 2F). In conclusion, PD-1 blockade in the PyMT tumor model was largely ineffective, whereas downregulation of BTLA surface expression reduced tumor growth and pulmonary metastasis, while it did not prevent the occurrence or outgrowth of new tumors.

### 2.3. BTLA Downregulation Increases NKT Cell Numbers

**Figure 3:**
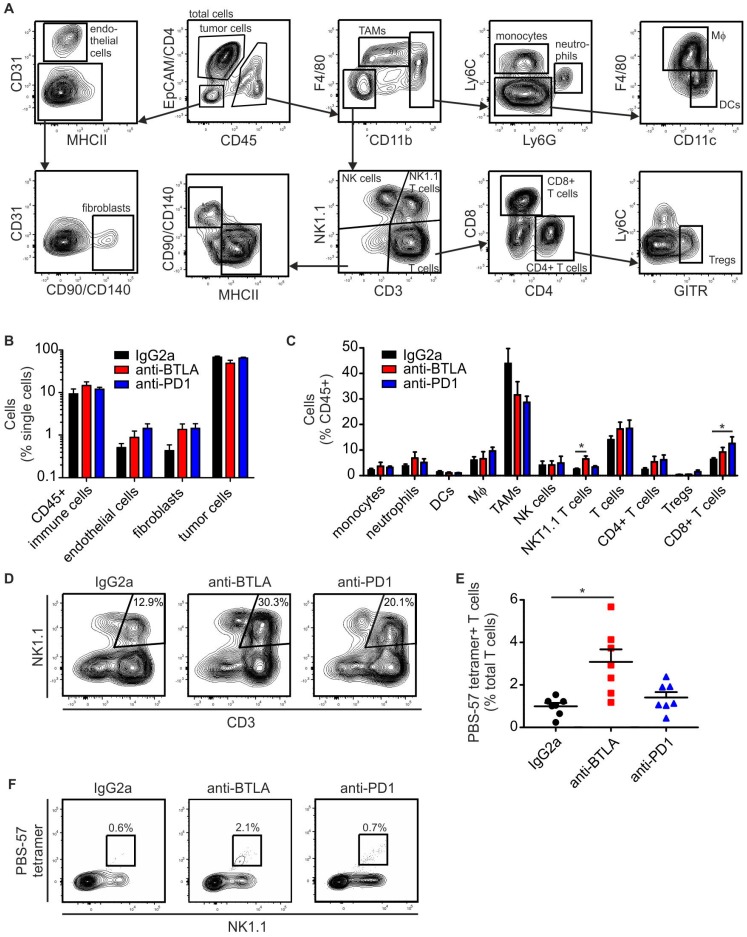
Anti-BTLA antibodies increase tumor infiltrating NKT cell numbers. PyMT tumors were treated with 5 mg/kg of each anti-BTLA, anti-PD-1 or isotype control (IgG2A) antibodies (*n* = 7 each) twice weekly for three weeks once the first tumor reached a size of 0.5 cm in diameter. Tumors were harvested for FACS analysis 21 days after initial treatment. (**A**) Representative FACS plots show gating strategy to identify cellular subsets in PyMT tumors. (**B**) The relative abundance of tumor cells, CD45+ immune cells, fibroblasts and endothelial cells in the total single cell population is shown. (**C**) The relative abundance of myeloid and lymphoid cell subsets within total CD45+ cells is displayed. (**D**,**E**) The graph indicates the relative abundance of PBS-57-CD1d tetramer reactive type I NKT cells. (**F**) Representative FACS plots indicate the expansion of PBS-57-CD1d tetramer reactive type I NKT cells upon anti-BTLA treatment. *p*-values were calculated using one-way ANOVA with Bonferroni’s correction, * *p* < 0.05.

**Figure 4:**
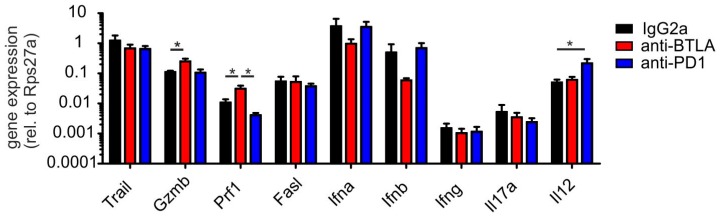
Anti-BTLA antibodies induce cytotoxic lymphocyte marker expression. PyMT tumors were treated with 5 mg/kg of each anti-BTLA, anti-PD-1 or isotype control (IgG2A) antibodies (*n* = 7 each) twice weekly for three weeks once the first tumor reached a size of 0.5 cm in diameter. Tumors were harvested for quantitative PCR analysis 21 days after initial treatment. The expression of the indicated genes relative to the house-keeping gene Rps27a is displayed. *p*-values were calculated using one-way ANOVA with Bonferroni’s correction, * *p* < 0.05.

To approach a mechanistic explanation for the efficacy of the anti-BTLA antibody, we probed the tumors for their cellular composition. We used a multi-spectral FACS approach that allows determining tumor cell and major stromal cell proportions simultaneously (Figure 3A). The proportions of tumor cells in comparison to major stromal cell subsets (CD45+ immune cells, fibroblasts, endothelial cells) were not significantly changed, although, as expected, there was a tendency of reduced tumor cell abundance in anti-BTLA antibody treated tumors (Figure 3B). When looking closer at individual immune cell subsets, we did not detect significant changes in myeloid cell populations. However, we observed alterations in lymphocyte subset abundance. Anti-PD-1 treatment significantly increased the number of CD8+ cells, indicating that PD-1 blockade was principally working (Figure 3C). BTLA blockade increased the number of NK1.1+ T cells (Figure 3C,D), and the number of PBS-57-loaded CD1d tetramer-recognizing type I NKT cells (Figure 3E,F). Next, we analyzed potential markers of anti-tumor immunity in PyMT tumors at the mRNA level. Anti-PD-1 antibody treatment selectively increased IL-12 levels, while anti-BTLA antibody treatment increased perforin and granzyme B expression, both being markers of cytotoxic cells (Figure 4). In conclusion, NKT cell numbers and the numbers of other T cells with cytotoxic potential increased upon downregulation of BTLA expression on the surface of NKT cells. This translated into reduced tumor growth. In contrast, anti-PD-1 treatment increased IL-12 expression and the relative abundance of CD8+ T cells, but did not significantly affect tumor growth. 

### 2.4. High BTLA and ZBTB16 Expression Defines a Subset of Breast Cancer Patients with Favorable Prognosis

**Figure 5:**
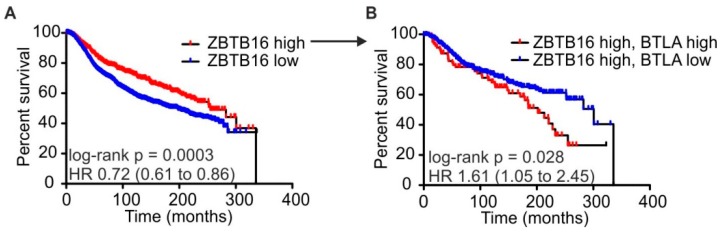
Combined BTLA and ZBTB16 expression predict mammary carcinoma patient survival. The METABRIC dataset (Curtis et al., 2012) was analyzed concerning a correlation of the NKT cells lineage determining transcription factor ZBTB16 and BTLA with overall survival. (**A**) Survival rates of patients expressing high (>75% percentile) or lower (<75% percentile) levels of ZBTB16. (**B**) Survival rates of patients expressing high (>75% percentile) levels of ZBTB16 after further separation into groups expressing high (>75% percentile) or lower (<75% percentile) levels of BTLA. *p*-values were calculated using log-rank test.

To investigate whether our findings might be relevant in human mammary carcinoma patients, we explored the published METABRIC dataset, containing gene expression and clinical data of ~ 2000 patients with mammary cancer [20]. BTLA expression did not predict patient survival per se (data not shown), but high expression of ZBTB16 (PLZF), a lineage-determining transcription factor of NKT cells, was associated with favorable overall survival (ZBTB16-high, mean survival 255.0 months; ZBTB16-low, mean survival 196.6 months) (Figure 5A). This indicates that a high number of infiltrating ZBTB16-expressing cells, such as NKT cells and γδ T cells, constitute a survival benefit for patients with breast cancer. Our findings from the PyMT model suggested that high BTLA expression on NKT cells was associated with tumor development. We therefore asked if high BTLA expression would limit the survival benefit resulting from high ZBTB16 expression. Indeed, when we separated patients expressing high ZBTB16 levels into BTLA-high versus BTLA-low groups, co-expression of high BTLA and ZBTB16 levels was associated with poor survival when compared to expression of high ZBTB16 combined with low BTLA levels (ZBTB16-high + BTLA-high, mean survival 201.8 months; ZBTB16-high + BTLA-low, mean survival 300.7 months) (Figure 5B). Thus, high BTLA expression limits the benefit of a strong ZBTB16-expressing cell infiltrate. Overall, these data suggest a possible role for BTLA in survival of patients with mammary carcinoma, at least under conditions of high innate-like lymphocyte infiltration.

## 3. Discussion

In our study, downregulating the expression of BTLA by a monoclonal antibody on tumor-infiltrating NKT cells induced protective immunity, likely due to the expansion of NK1.1+ T cells, as well as, or including, type I NKT cells. Expression of the gene encoding NK1.1, KLRB1, was identified as the strongest positive prognostic marker for survival across a panel of solid tumors [1]. In mouse strains expressing NK1.1, NK1.1+ T cells include, besides type I NKT cells, activated CD8+ T cells and γδ T cells [21,22]. All of these T cell subsets have cytotoxic potential, which may explain the upregulation of cytotoxic cell markers at mRNA level upon anti-BTLA antibody administration. It remains to be tested which of the mentioned T cell subsets contribute mainly to the observed tumor control upon anti-BTLA treatment. Interestingly ZBTB16 or PLZF is not only expressed by NKT cells, but also controls the development of other innate lymphocytes, including γδ T cells [23]. Moreover, BTLA expression was recently shown to block γδ T cell cytokine production and to limit γδ T cell-dependent dermatitis [24]. Thus, it remains to be determined which innate lymphocyte subset produces the anti-BTLA effect in mice, and which cellular subset mediates the association of ZBTB16 with patient survival in human mammary carcinoma. Future studies employing CD1d-deficient mice in the PyMT background or antibodies depleting specific cell subsets together with BTLA down-modulation may resolve this question, at least in mice.

To our surprise, anti-PD-1 treatment, besides inducing markers of anti-tumor immunity such as an increase in CD8+ T cells and IL-12 expression within the tumor, did not alter tumor development in the PyMT model. In a study using orthotopic transplantation of a PyMT-derived cell line, combined PD-1 and PD-L1 blockade did not affect tumor growth, although it reduced pulmonary metastasis in this more acute setting [25]. Markers of anti-tumor immunity were not evaluated in this study. Therefore, we do not know whether an increase in CD8+ T cells would have occurred as well. It is difficult to speculate why an increase in CD8+ T cells did not translate into protective immunity. One possibility would be that other suppressive mechanisms are preserved, including the expression of alternative immune checkpoints, or the absence of CD4+ T cell help [2,8].

We propose a major role for NKT cells in our model, since these cells selectively expressed high BTLA levels in PyMT mammary carcinoma. Moreover, BTLA has been prominently connected to activation of type I NKT cells before. Lack of BTLA expression on hepatic NKT cells increased hepatic inflammation in two independent studies [10,26].

Activity of type I NKT cells would require CD1d expression as well as the presentation of a specific lipid antigen. While CD1d is abundantly expressed by immune cells and tumor cells in the PyMT model (data not shown), production of a stimulatory type I NKT lipid antigen in mammary carcinoma is not described. In mice and humans, few endogenous type I NKT lipid antigens are known. Murine immune cells were recently shown to produce low levels of the prototypic exogenous lipid antigens, α-glycosylceramides including α-GalCer [27]. These low levels of α-glycosylceramides appear to be maintained by continuous degradation via α-galactosidase and acid ceramidase, which is necessary to prevent auto-reactivity [27]. Future studies might address expression levels of these enzymes and levels of α-glycosylceramides in breast tumors in comparison to non-transformed tissues. Besides α-glycosylceramides, specific lysophospholipids may represent potent endogenous type I NKT cell lipid antigens. Lysophosphatidylcholine was shown to activate human type I NKT cells [28], even in a tumor context [29]. Moreover, ether lysophosphatidylethanolamine and potentially other ether lipids were identified as self-lipid antigens for type I NKT cells [30]. Interestingly, anabolic pathways to generate ether lipid per se were upregulated in tumors, including breast tumors [31]. Whether increased ether lipids synthesis in tumors provides endogenous NKT cell lipid antigens is, however, unclear. Future studies might combine BTLA depletion with NKT cell activation protocols, including supplementation of α-GalCer or inhibition of enzymes degrading endogenous type I NKT cell ligands. Moreover, further studies are needed to investigate the functional phenotype of type I NKT cells (and other NK1.1+ T cells) upon BTLA downregulation.

When considering a potential inhibitory effect of BTLA on NKT cell activation, the question remains which cell in the tumor microenvironment expresses its ligand HVEM. This is of interest to conduct future functional studies concerning downstream effects of BTLA blockade in NKT cells. We observed expression of HVEM on most cells in the tumor microenvironment, including tumor cells. Therefore, it would be necessary to investigate the spatial relationship of NKT cells with other cells in the tumor microenvironment. Such studies, which require detection of type I NKT cells via immunohistochemistry, are limited by the lack of specific type I NKT cell-recognizing antibodies in the mouse. Moreover, interaction of BTLA with HVEM can occur in *cis*, i.e., on the same cell. Future studies might therefore rely on isolation and in-depth characterization of type I NKT directly from PyMT tumors, before and after anti-BTLA treatment.

One might consider whether targeting HVEM instead of BTLA in the tumor microenvironment produces similar beneficial effects concerning tumor growth, or be even superior based on its abundant expression. HVEM binds to other lymphocyte surface molecules besides BTLA, namely lymphotoxin-α (LTα) and tumor necrosis factor superfamily member 14 (TNFSF14, LIGHT). In contrast to the co-inhibitory interaction with BTLA, coupling of HVEM to LTα or LIGHT produces a co-stimulatory signal, enhancing lymphocyte activation [32]. BTLA blockade therefore may serve two purposes at once. It releases a brake on lymphocyte activation, while simultaneously allowing stimulatory signals to be effective.

Finally, immune checkpoint blockade needs to consider potential auto-immune side-effects that would limit its clinical applicability. Since BTLA is highly expressed on B cells and global BTLA deletion was associated with enhanced auto-immune reactions in a number of studies [3,10,26,33], caution is warranted when envisioning anti-BTLA antibodies for the treatment of cancer in humans. However, as opposed to global deletion of BTLA in the germline, therapeutic blockade of the HVEM/BTLA pathway did not alter alloantibody production in a model of allograft rejection [34]. Moreover, no apparent auto-immune disease developed in mice treated with anti-BTLA antibodies in our study during the course of three weeks. Thus, while further investigation is certainly needed, BTLA down-modulation might be of interest to increase the activity of innate-like cells in tumors.

## 4. Materials and Methods

### 4.1. Animal Experiments

Mice expressing the polyoma virus middle T oncoprotein (PyMT) under the Mouse Mammary Tumor Virus (MMTV) promoter in a C57BL/6 background were used. In the PyMT model, the expression of the PyMT oncoprotein is restricted to the mammary epithelium, which results in the appearance of mammary tumors starting from 6 weeks after birth in C57BL/6 mice and the occurrence of pulmonary metastases starting after 18 weeks [35]. For analysis of immune checkpoint receptor expression, tumor bearing PyMT mice were sacrificed 18 weeks after birth, perfused with PBS and tumors were harvested for downstream applications. For immune checkpoint blockade in a therapeutic setting, animals were either treated with 5 mg/kg anti-BTLA antibody (clone 4G12b, produced as described before [34]), anti-PD-1 antibody (clone RMP1-14) or a rat IgG2a isotype control (both from BioXCell) once the first mammary tumor reached a size of 0.5 cm in diameter. Treatment with the antibodies was performed twice weekly for three weeks in regular intervals. Afterwards, animals were perfused and PyMT tumors and lungs were harvested for downstream applications. For all animal experiments the guidelines of the Hessian animal care and use committee were followed.

### 4.2. Immunohistochemistry

PyMT lungs were Zn-fixed and paraffin-embedded. For analyzing lung metastasis, lung sections were de-paraffinized, stained with Mayer’s hemalum (Merck KGaA), and examined under an Axioskop 40 (Zeiss) microscope. At least 9 independent sections of 3 different lung areas were analyzed.

### 4.3. Flow Cytometry 

Characterization of immune cell subsets in tumors was performed essentially as described before [16]. Tumor single suspensions were generated using the gentleMACS dissociator and the mouse Tumor dissociation kit (both from Miltenyi Biotec). Single cell suspensions were stained with fluorochrome-conjugated antibodies and analyzed on a LSRII/Fortessa flow cytometer (BD Biosciences). Data were analyzed using FlowJo software Vx (Treestar). Antibodies and secondary reagents were titrated to determine optimal concentrations. CompBeads (BD) were used for single-color compensation to create multi-color compensation matrices. For gating, fluorescence minus one (FMO) controls were used. The instrument calibration was controlled daily using Cytometer Setup and Tracking beads (BD). The following antibodies were used: anti-CD3-PE-CF594, anti-CD4-BV711, anti-CD8-BV650, anti-CD11c-AlexaFluor700, anti-CD140-PE, anti-CD326-BV711, anti-Ly6C-PerCP-Cy5.5, anti-NK1.1-BV510 (BD Biosciences), anti-CD31-PE-Cy7 (eBioscience), anti-CD45-VioBlue, anti-CD90.2-PE, anti-MHC-II-APC (Miltenyi Biotec), anti-CD11b-BV605, anti-F4/80-PE-Cy7, anti-GITR-FITC, anti-Ly6G-APC-Cy7, and anti-γδTCR-APC (Biolegend). 7-AAD was used for dead cell exclusion. Immune checkpoint expression on T cells was analyzed using the following antibodies: anti-CD3-PE-Cy5, anti-CD4-BV711, anti-CD8-BV650, anti-CD19-APC-Cy7, anti-CD45-AlexaFluor700, anti-CD69-PerCP-Cy5.5, anti-NK1.1-BV510 (BD Biosciences), anti-CD272 (BTLA)-PE, anti-CD278 (ICOS)-FITC, anti-CD279 (PD-1)-BV421 (Miltenyi Biotec), anti-CD11b-BV605 (BioLegend), and in-house generated PBS-57-loaded CD1d tetramer-PE-CF594 (see below). HVEM expression was analyzed using anti-HVEM-PE (Biolegend).

### 4.4. Generation of CD1d Tetramers

To identify type I NKT cells, CD1d tetramers loaded with the α-GalCer analogue PBS-57 were used. PBS-57-loaded, biotinylated murine CD1d monomers were obtained through the NIH Tetramer Core Facility. Tetramers were generated with PE-CF594-strepavidin (BD Biosciences) using standard procedures as suggested by the NIH Tetramer Core Facility.

### 4.5. RNA Isolation and RT-qPCR

RNA was isolated using the PeqGold^®^ protocol (Peqlab Biotechnologie, Erlangen, Germany). RNA was transcribed into cDNA using Fermentas’ reverse transcriptase Kit (ThermoFisher Scientific). Real-time qPCR was performed using the MyIQ real-time PCR system and SYBR green (both from Bio-Rad, Munich, Germany). The following primers were used from Biomers (Ulm, Germany): mouse *Rps27a* sense 5′-GACCCTTACGGGGAAAACCAT-3′, antisense 5′-AGACAAAGTCCGGCCATCTTC-3′. For all other targets, QuantiTect Primer Assays (Qiagen) were used.

### 4.6. Statistical Analysis

Data were analyzed using GraphPad Prism 5.0 (GraphPad Software, San Diego, USA). *p-*values were calculated using two-tailed Student’s *t* test, one-way ANOVA or two-way ANOVA. To check for Gaussian distribution, D’Agostino and Pearson omnibus normality tests were performed. Parametric or non-parametric tests were applied accordingly. Asterisks indicate significant differences between experimental groups (* *p* < 0.05, ** *p* < 0.01, and *** *p* < 0.001).
